# Editorial: Noncommunicable diseases and mental health experiences before and after the COVID-19 pandemic

**DOI:** 10.3389/fpubh.2023.1177869

**Published:** 2023-03-21

**Authors:** Rahul Shidhaye, Annika C. Sweetland, Jerome T. Galea, Mir Nabila Ashraf, Malay K. Mridha, Hannah Maria Jennings, Aliya Naheed

**Affiliations:** ^1^Department of Psychiatry, Pravara Institute of Medical Sciences Ahmednagar, Ahmednagar, India; ^2^Department of Health, Ethics, and Society, Care and Public Health Research Institute, Maastricht University, Maastricht, Netherlands; ^3^Department of Psychiatry, Columbia Vagelos College of Physicians and Surgeons/New York State Psychiatric Institute, New York, NY, United States; ^4^School of Social Work, University of South Florida, Tampa, FL, United States; ^5^Health Systems and Population Studies Division, Initiative for Non Communicable Diseases, International Centre for Diarrhoeal Disease Research Bangladesh, Dhaka, Bangladesh; ^6^Center for Non-communicable Diseases and Nutrition, James P Grant School of Public Health, BRAC University, Dhaka, Bangladesh; ^7^Department of Health Sciences, University of York, York, United Kingdom; ^8^Hull York Medical School, York, United Kingdom

**Keywords:** non-communicable diseases, mental health, COVID-19, comorbidity, health services

Globally, Non-Communicable Diseases (NCDs) such as cardiovascular disease (CVD), diabetes, cancer, and respiratory diseases account for approximately 71% of deaths (1). NCDs disproportionately affect people in Low- and Middle-Income Countries (LMICs) and cause the death of approximately 32 million people per year. Mental illness is another global burden that accounts for around 32.4% of years lived with disability and 13% of disability-adjusted life-years (DALYs) (2).

Additionally, the current COVID-19 pandemic has severe repercussions for people with NCDs (3). Evidence suggests that NCDs such as hypertension and type 2 diabetes are found to be the more frequent co-morbidities with SARS-CoV-2 that require intensive care (4). However, our understanding of the impact of COVID-19 on major NCDs and the depth of evidence regarding an impactful solution to mitigate the burden of NCDs amid the COVID-19 situation is limited.

The aim of this Research Topic was to generate evidence gathered both before and during the COVID-19 pandemic that includes conceptual, epidemiological, intervention, and policy papers to address the related issues of noncommunicable disease (hypertension, diabetes, COPD, etc.) and mental health globally.

The Research Topic includes 13 contributions from 96 authors presenting their research conducted in different parts of the world. The maximum number of papers are from China (*n* = 4), followed by Mozambique (*n* = 2), and one paper each from Bangladesh, Colombia, Ethiopia, Lebanon, Panama, South Korea, and The Netherlands. Mental health comorbidity in patients with pre-existing NCDs during the COVID-19 pandemic is assessed in four papers, one paper deals with the quality-of-care, and the impact of COVID-19 on mental health at population level is presented in four papers. Two papers are about factors affecting access to mental health care and the remaining two papers deal with the consequences of COVID-19 on the mental health of Health Care Providers (HCPs). Below we provide an overview of all the papers included.

## COVID-19 and multimorbidity

Mental health of individuals with pre-existing NCDs was severely affected during COVID-19. Amin et al. describe a high prevalence of depression and anxiety among patients who were hospitalized for heart disease during the pandemic in Dhaka, Bangladesh. Their findings underline the importance of mental health screening of patients with Cardio-Vascular Diseases in order to offer sufficient support. An online cross-sectional survey conducted during April–May 2022 of Chinese women receiving oral chemotherapy for ovarian cancer by Mao et al. found that a higher proportion of these women reported anxiety symptoms and lower quality of life. This could possibly be due to the COVID-19 pandemic situation and the resultant delays in receiving care according to the authors. Similarly, Wang et al. report a high proportion of Chinese patients with advanced melanoma having anxiety, depression, and importantly the fear of progression of cancer.

The impact of COVID-19 on the quality of life of patients with chronic NCDs in Ethiopia is presented by Ayalew et al.. Their findings suggest that female gender and presence of a common mental disorder led to poorer quality of life (QoL) in patients with chronic NCDs. The study also highlights another important finding that lower educational status is significantly associated with better QoL specially during the pandemic which is supported by other studies as well. Determinants of quality-of-care provided to patients with diabetes and hypertension in a fragile context (Lebanon) is described by Saleh et al.. As this study began just before the pandemic started, it captures the impact of COVID-19 on NCD care provision.

## COVID-19 and mental health

The fear of contracting COVID-19 and the measures undertaken to curb the transmission of the infection had a significant impact on the mental health of the population. Analysis of the data from a large Korean Community Health Survey by Han et al. indicates a high level of COVID-19 related anxiety and a negative impact on physical activity, diet, and sleep pattern. Similar psychological response to the pandemic is reported from the diametrically opposite part of the globe, Panama. Oviedo et al. present the findings from a community survey of adults in which, as in the Korean study, they report high levels of stress, anxiety, and depression among a community sample of adults. School/college closure due to COVID-19 affected mental health of children and adolescents. Li describes the role of various risk factors associated with depression and anxiety in college students from Shanghai, China.

Knowledge of the mental health conditions (commonly known as mental health literacy) and perception related to mental health care provision play an important role in access to mental health care. Li et al. explore mental health literacy using a cross-sectional community survey of the residents from Jiangsu province in China during the subsequent waves of the pandemic. To improve the perception of ease to access mental health care, the need for better communication between the State health agencies, health care providers, and the patients with mental health conditions is highlighted by Gómez-Restrepo et al..

Attitude of HCPs toward mental health conditions also plays a role in care provision. Using an explanatory sequential mixed-methods study design, Mandlate et al. discuss knowledge and attitudes about mental health among lay counselors in Mozambique.

## COVID-19 and health care providers

Being on the frontline, HCPs were severely affected physically, psychologically, and socially due to the COVID-19 situation. Czepiel et al. present the findings on an online survey of Dutch HCPs highlighting the subjective experience and mental health conditions reported by these HCPs. Interesting findings from Mozambique are reported by Feliciano et al. as the HCPs there reported a reduction in burnout, which the authors attribute to reduced caseloads during lockdown.

## Challenges and recommendations

This Editorial Board was formed by reviewers from both High and low-and middle-income countries. Despite the huge pool of reviewers and the efficient process of the Frontiers to invite reviewers, one of the challenges we faced was to identify potential reviewers. We highly recommend time and opportunity to create a pool of reviewers, particularly from Low- and Middle-income countries and conduct workshops for capacity building of the young reviewers who can contribute effectively to reviewing the manuscripts in a timely manner.

In the conclusion, this Research Topic has provided an opportunity for researchers to showcase their innovative approach to modern thinking in publishing research, particularly meeting the demand of disseminating new knowledge during the COVID-19 pandemic. More topic areas should be included encouraging the writers to adopt a new way of presenting ideas of thinking critically about how the research evidence compares with pre-COVID vs. post-COVID settings and would be useful to policymakers for adopting rapid solutions in tackling NCD and mental health in a public health crisis such as COVID-19.

## Author contributions

AN initiated the Research Topic. RS, AS, JG, MA, MM, HJ, and AN were the topic editors. RS wrote the first draft. All authors contributed to the manuscript revision, read, and approved the final version.
